# New Process for the Detection of Copper, Applicable to Medico-legal Analysis

**Published:** 1842-03

**Authors:** 

**Affiliations:** Lyons


*New Process for the Detection of Copper, applicable to Medico-legal Analysis.* By M. Verguin, of Lyons.—This process was suggested by an observation made some years since in analysing a mineral of copper. By accident, I placed the solution in a platinum capsule, and wishing to estimate the copper in a metallic state, placed therein a plate of iron. While the iron was not in contact with the platinum, no action took place; but the instant it came in contact with this latter, the capsule was covered with a very adherent film of copper, no precipitation taking place on the iron. The adhesion was so strong, that to separate them, nitric acid had to be used. I paid but little attention to this fact, and had almost forgotten it, when it was called to mind by reading the process of Dr. Christison for the detection of mercury; and I sought out a simple process which might be applied to the determination of copper in medico-legal analysis. It is this which is the subject of the present note.

Before entering on the description of my process, I will rapidly examine the different reagents employed—their degree of certainty, and the case in which they will be insufficient. These reagents are ammonia, the yellow cyanide of potassium and iron, and metallic iron.

Ammonia acts by dissolving the oxide of copper, and producing a fine blue color; this color is with difficulty perceived, if—1st, the liquid tested contains a salt of which the base is precipitated by the ammonia, for then it is disguised by the precipitate; 2d, if it is colored by organic matter. It is true we may filter and decolorise by animal charcoal—but when but little of the substance is possessed, it is very important not to lose any by the number of manipulations.

The cyanide of potassium and iron detects minute quantities of copper; for this it is necessary that the liquid should be pure, and especially that it should not contain a trace of iron, for without this it is impossible to distinguish the brown color of the salt of copper, when mixed with the blue of the salt of iron.

Iron acts by decomposing the salt of copper and precipitates the copper in a metallic state, an action well represented by the following formula: $(Cu3S) + Fe = Cu + (Fe3S)$, where we may see that the iron takes the place of the copper, and when the reaction terminates, we have sulphate of iron and metallic copper. But it is necessary that the liquid should be acidulated by a little acid; but if too much be added and the copper be in small amount, the iron blackens, and prevents the copper being readily distinguished. Moreover the copper does not adhere, and the least disturbance causes it to separate.

These uncertainties do not exist in the process which I am about to describe, and which is no more than the application of the observation of which I spoke in the commencement.

It is proper that the liquid to be examined, if it be dilute, should be somewhat concentrated, and slightly acidulated with hydrochloric acid; a drop is to be placed on a plate of platinum, and covered with a well polished plate of iron, in such a manner that the iron shall touch both the liquid and platinum. In a few seconds the platinum exhibits a very adherent covering of copper throughout every part touched by the liquid. The explanation of this fact depends entirely upon electro-chemical theory; it results from principles which I shall rapidly enumerate: 1st, when two metals are placed in contact, there is a production of electricity, one of the metals being positively and the other negatively electrified. 2d, when a solution is subjected to the action of the pile, the salt is decomposed, the acid passing to the positive, and the base to the negative pole. There are some salts which are not only thus decomposed into acid and base, but still further the base itself is decomposed into metal and oxygen; in this case the metal only passes to the negative pole, the oxygen appearing at the positive pole.

But, when iron and platinum are placed in contact, there is a developement of electricity, which developement is the more active when a saline solution is present; the iron becomes positive, the platinum negative. The salts of copper have the property of being decomposed not only into acid and base, but into oxygen and metal. Hence the acid being more oxygenated is transferred to the iron or positive pole, while the metal is fixed on the platinum or negative pole.

Such is the process which I offer as most exact, and not liable to the uncertainties of the other methods. It is simple, does not require any manipulation which may not be performed by persons with but a slight chemical knowledge, and I believe will be found useful in medico-legal experiments.—*American Journal of Pharmacy* from *the Jour. de Pharm.*

